# Prevalence and characteristics of sex trafficking among youth experiencing homelessness: A cross-sectional study

**DOI:** 10.1371/journal.pone.0357091

**Published:** 2026-08-26

**Authors:** Higinio Fernández-Sánchez, Jennifer Jones, Utsav Arandhara, Nikhil Padhye, Adeline Nyamathi, Marguerita Lightfoot, Yasmeen Quadri, Mary Paul, Michael S. Businelle, Diane Santa Maria

**Affiliations:** 1 Center for Nursing Research, Cizik School of Nursing, University of Texas Health Science Center at Houston, Houston, Texas, United States of America; 2 Sue & Bill Gross School of Nursing, University of California Irvine, Irvine, California, United States of America; 3 Oregon Health & Science University-Portland State University School of Public Health, Portland, Oregon, United States of America; 4 Department of Family and Community Medicine, Baylor College of Medicine, Houston, Texas, United States of America; 5 Department of Pediatrics, Baylor College of Medicine, Houston, Texas, United States of America; 6 TSET Health Promotion Research Center, University of Oklahoma Health Sciences Center (OUHSC), Oklahoma City, Oklahoma, United States of America; University of New Mexico Health Sciences Center, UNITED STATES OF AMERICA

## Abstract

**Background:**

Youth experiencing homelessness (YEH) face elevated risk for sex trafficking due to interconnecting structural, interpersonal, and psychological vulnerabilities. Despite growing recognition of this link, regional data from the southern United States remain limited. This study examines the proportion of YEH reporting experiences consistent with sex trafficking and describes associated characteristics among a sample of YEH in Houston, TX.

**Methods:**

Baseline data (September 2019–May 2023) from a randomized controlled trial of a nurse-led HIV prevention intervention were analyzed. Participants (N = 450; ages 16–25) completed self-administered surveys. Sex trafficking was defined as endorsing ≥1 of five Commercial Sexual Exploitation items from the Urban Institute Human Trafficking Screening Tool. Adjusted and unadjusted modified Poisson regression models with robust variance estimation were used to examine relationships between sex trafficking and age, gender, race/ethnicity, sexual orientation, foster care history, past-30-day drug use, and last permanent residence in Houston.

**Results:**

Within this sample of YEH, 23.1% (104/450) reported experiences consistent with sex trafficking. In adjusted models, those who identified as female vs. male (aPR = 2.341, 95% CI: 1.530–3.582) were more likely to report being trafficked.

**Conclusions:**

Among YEH in Houston, study findings showed that trafficking disproportionately impacted those who identify as female. Findings suggest a need for routine, trauma-informed trafficking screening; service strategies to reach impacted youth; and longitudinal research to clarify causal pathways and guide tailored prevention and care for diverse youth populations.

**Trial registration:**

ClinicalTrials.gov NCT03910218

## Introduction

At any given time, an estimated 6.3 million people are sex trafficked globally [1]. In the United States (U.S.), sex trafficking is defined by the Trafficking Victims Protection Act of 2000 as *“the recruitment, harboring, transportation, provision, obtaining, patronizing, or soliciting of a person for a commercial sex act* [2]*.”* Notably, the elements of force, fraud, or coercion must be present when the commercial sex act involves an adult; however, force, fraud, or coercion is not required if a minor is involved in the commercial sex act [2]. The number of individuals who have been sex trafficked in the U.S. is unknown. Available data typically combines labor and sex trafficking, and is restricted to hotline reports, arrests, and legal proceedings, which encompass only a fraction of the problem. One example is the National Human Trafficking Hotline annual summary, which shares national and state-specific data on all communications to provide a glimpse into the number of individuals who may be trafficked in the U.S. The annual summary reports: 1) signals, which are communications received via a hotline call, online report, web chat, SMS, or email; 2) cases, which represent a distinct situation that can involve one or more potential victims of trafficking reported to the hotline through one or more signals; and 3) victims, which are individuals involved in one or more cases. The National Human Trafficking Hotline also counts the tips received about potential situations of sex and/or labor trafficking from signals, as opposed to signals requesting information and resources. The latest available summary from the National Human Trafficking Hotline for 2024 reported receiving 8,024 signals from victims or survivors of human trafficking, 11,999 cases were identified, and 21,865 victims were involved in these cases [3]. Texas had the second-highest percentage of total signals (7.5%) and total cases (11.3%) next to California [3]. In 2024, the hotline received 1,094 tips about potential situations involving trafficking in Texas, and 68% of those involved sex trafficking [3]. Similar data for more specific regions or cities within Texas do not exist, however, one report estimated that there were over 14,500 victims in the greater Houston area in 2019 [4]. Data from law enforcement response and human trafficking cases in federal court, indicate that age and gender are factors contributing to vulnerability, as female youth and young adults comprise most sex trafficking victims [5,6]. Available sources can provide insight into potential victims and survivors of sex trafficking, or those that make it to the court system but none provide an estimated prevalence in general or subpopulations. It is critical to better understand the problem in a large urban area like Houston, Texas among the most vulnerable populations.

In the 2024 U.S. Department of Housing and Urban Development (HUD) Annual Homeless Assessment Report, the point-in-time count estimated that there were over 38,000 unaccompanied youth on a single night in the U.S [7]. The McKinney-Vento Act of 1987 provides the foundation for the definition of homelessness among youth or young adults, which includes those “who lack a fixed, regular and adequate nighttime residence” as well as those sharing housing with other persons (doubled-up), living in temporary accommodating such as motels, places not designed for sleeping, and migratory children [8]. The application of this definition has changed over time. While the HUD narrowed the definition by restricting individuals “doubled up” for housing eligibility purposes (inclusive of the HEARTH Act amendments) [9], the Department of Education retains a broad definition to identify and support students in schools. Generally, the definition used by the Department of Education reflects a broader spectrum of housing instability among youth and is more likely to capture youth who may need a variety of supportive services. Youth who are unstably housed tend to lack basic needs such as food, shelter, and protection, making them more vulnerable to sexual exploitation, which legally constitutes sex trafficking when the individuals involved are minors [10–13]. Numerous studies have documented sex trafficking victimization rates between 11–41% among youth experiencing homelessness (YEH) across various U.S. regions, including Atlanta, Kentucky, and Arizona [10,14,15]. Efforts to develop and deploy consistent trauma-informed screening tools for YEH are ongoing but are not yet widely implemented [16,17].

YEH face elevated risk for sex trafficking due to intersecting structural and interpersonal vulnerabilities. [14]. Histories of abuse, family conflict, foster care involvement, housing instability, and adverse childhood experiences have all been associated with increased vulnerability to sexual exploitation and trafficking [14,18]. Traffickers often exploit unmet needs for safety, housing, emotional support, and financial resources, while limited access to trauma-informed services may further increase risk and reduce opportunities for intervention. [13,14]. These findings are consistent with evidence from migrant populations, where traffickers exploit housing instability, economic hardship, immigration-related fears, and social marginalization to maintain control over victims [19].

Despite increasing recognition of the link between homelessness and sex trafficking, significant gaps remain in understanding the prevalence and specific characteristics of trafficking survivorship among YEH. Moreover, few studies have disaggregated findings to examine the diverse experiences of youth based on race, gender, foster care system involvement, and substance use. This lack of nuanced data hinders the development of tailored prevention interventions and policies that can address the complex realities of trafficked youth. To address this gap, the current study examined the prevalence and characteristics of sex trafficking among a sample of YEH in Houston, Texas, a city with high reported rates of trafficking and a large, diverse unhoused youth population. By examining demographics, experiential factors, and behavioral factors, this study aimed to inform trauma-informed, accessible approaches to prevention and care [20].

## Methods

### Study design and setting

We employed a cross-sectional design to examine the prevalence and characteristics of sex trafficking among YEH in Houston, Texas. The design allowed for the identification of group differences between those who experienced sex trafficking and those who did not and facilitated the exploration of potential associations between trafficking and demographic, experiential, and behavioral factors. Data were drawn from the baseline assessment of a large randomized controlled trial (RCT) evaluating the efficacy of a nurse-led HIV prevention intervention for YEH [21,22]. The trial was conducted across six community-based sites that provide services to YEH in the Houston metropolitan area between September 01, 2019, and May 31, 2023. The current analyses used baseline data collected before intervention exposure, which allowed for an independent cross-sectional examination of key variables unrelated to the intervention outcomes.

### Participants

A total of 450 YEH were enrolled in the baseline phase of the RCT and were included in the current cross-sectional analyses. Eligibility criteria for participation was determined by trained study staff using a screener to assess for the following criteria: (1) being between the ages of 16 and 25 years; (2) currently experiencing homelessness as defined by residing in a shelter, a temporary location including a hotel, motel, or couch surfing, a place not meant for habitation including outside, abandoned building, bus/train station, or car last night; and (3) able to read and speak English. Definition of homelessness was based on the 1987 McKinney-Vento Homeless Assistance Act criteria to capture the full continuum of housing instability among youth. Full eligibility criteria and trial methodology are described in detail elsewhere [21]. Participants were recruited for the RCT using convenience and snowball sampling from shelters and drop-in centers serving YEH, and enrollment was conducted by the research coordinator, research assistants, and research nurses. For the current analyses, participants were excluded if they did not respond to all five questions assessing commercial sex trafficking experiences. Because experiencing sex trafficking was defined as reporting one or more affirmative responses across the full set of trafficking indicators, excluding incomplete responses reduced the potential for outcome misclassification and ensured consistent application of the study definition across participants.

### Data collection

Data were collected via self-administered digital surveys during the baseline assessment of the RCT. Participants completed the survey in a private setting using either an iPad provided by study staff or their personal device, with trained research staff available to assist if needed and to ensure privacy. The survey included validated items and standardized measures assessing sociodemographic characteristics, prior systems involvement, psychosocial factors, substance use, and experiences of commercial sexual exploitation. Due to the COVID pandemic, which occurred during the study, participants were given the option to complete surveys remotely on their devices and informed that they could call the staff if they needed assistance.

### Instruments

#### Sex trafficking.

Sex trafficking was assessed using five items from the Commercial Sexual Exploitation (CSE) domain of the Human Trafficking Screening Tool developed by the Urban Institute [16]. These items asked participants whether they had ever experienced any of the following at their place of employment: being forced to perform sexual acts they were uncomfortable with; having their photo posted online to solicit clients for sex; being forced to engage in sexual acts with family, friends, or business associates in exchange for money or favors; being encouraged or pressured to engage in sexual acts, including taking sexual photos or videos; or being forced to trade sex for money, shelter, food, or other necessities through online websites, escort services, street-based prostitution, or other venues. Response options for each item were “yes,” “no,” or “skip.” Participants who answered “yes” to any of the five items were categorized as experiencing sex trafficking, while those who answered “no” to all five items were classified as not experiencing trafficking.

### Sociodemographic characteristics

Age, ethnicity/race, gender identity, and sexual orientation were assessed using single-item questions with categorical response options.

### History of foster care

History of foster care was measured by asking participants, “Have you ever been in foster care?” with response options of “yes” or “no.”

### Substance use

Participants indicated whether they had used any of the following substances in the past 30 days (yes/no): marijuana/hashish; hallucinogens (LSD, PCP, psychedelics, mushrooms); inhalants; crack/freebase; heroin and cocaine together (speedball); cocaine (by itself); heroin (by itself); street methadone; alcohol, amphetamines or other stimulants; tranquilizers, barbiturates, or sedatives; or other drugs not listed.

### Sexual assault

Sexual assault was captured with one question asking if the participant had ever had sex against their will. Response options were “yes” or “no.”

### Houston residence

To determine whether participants were previously stably housed in Houston or if they came from another area, participants were asked if their last permanent home was in the Houston area. Response options were “yes” or “no.”

### Ethical considerations

All participants in the study signed a consent form and were informed that they could refuse to answer any questions in the baseline survey and stop taking the survey at any time. The requirement of parental consent for unaccompanied minors was waived by IRB because they met the federal criteria for a waiver of parental permission when such requirements are unreasonable or not feasible. Participants received a $20 gift card as compensation for their time to take the survey. Ethics approval was obtained from the Committee for the Protection of Human Subjects at The University of Texas Health Science Center Houston (HSC-SN-18-0993).

### Data analysis

The objective of this study was to determine the factors associated with the history/experience of sex trafficking among YEH. We assessed whether age, ethnicity/race, gender, sexual orientation, history of foster care, drug use and Houston residency were associated with experiencing sex trafficking. Missing data for all the variables were handled using the *mice* library package in R. A total of 10 datasets were imputed using variable appropriate parameters to provide a robust framework for regression analysis. The choice of multiple imputation was dictated by the fact that it uses the distribution of the available variables/data to impute the missing values, thereby preserving the structure and variability of the original data [23]. Multiple imputation also helped stabilize the effect of the “Skip” option in the outcome variable. Each ‘skip’ was considered as missing, and then the imputation algorithm was implemented to impute those results by keeping the distribution of the other variables in the data set fixed. Independent variables were selected a priori based on prior literature demonstrating associations between sex trafficking and demographic characteristics, foster care involvement, substance use, sexual orientation, and housing-related factors among YEH. The analysis was exploratory and intended to identify correlates rather than develop a predictive model.

Descriptive statistics were used to summarize the overall distribution of the demographic variables. Modified Poisson regression was used for assessing associations of sex trafficking with age, gender, race/ethnicity, sexual orientation, foster care history, past-30-day drug use, and last permanent residence in Houston. Subsequently, multivariable modified Poisson regression was performed to assess the adjusted associations of predictors with sex trafficking. Statistical significance was assessed using the conventional type I error level of 0.05. Additionally, adjusted Prevalence Ratios (aPR) and corresponding 95% CI were reported to emphasize the effect sizes. The data analysis was carried out in R version 4.2.2.

## Results

### Demographics

The median age of participants was found to be 21 years (interquartile range = 4; 19–23 years). In this sample, 23.1% reported experiencing sex trafficking. Table 1 describes the responses to each of the five items from the Commercial Sexual Exploitation (CSE) domain of the Human Trafficking Screening Tool.

**Table 1:** Sex trafficking experiences (N = 450).

Experienced at place of employment	Yes	No
Forced to do something sexually that you didn’t feel comfortable doing	67 (14.9%)	383 (85.1%)
Put photo on the Internet to find clients to trade sex with	41 (9.2%)	409 (90.8%)
Forced to engage in sexual acts with family, friends, or business associates for money or favors	49 (10.9%)	401 (89.1%)
Encouraged or pressured to do sexual acts or have sex, including taking sexual photos or videos	67 (14.9%)	383 (85.1%)
Forced to trade sex for money, shelter, food or anything else through online websites, escort services, street prostitution, informal arrangements, brothels, fake massage businesses or strip clubs	48 (10.7%)	402 (89.3%)

**Fig 1:**
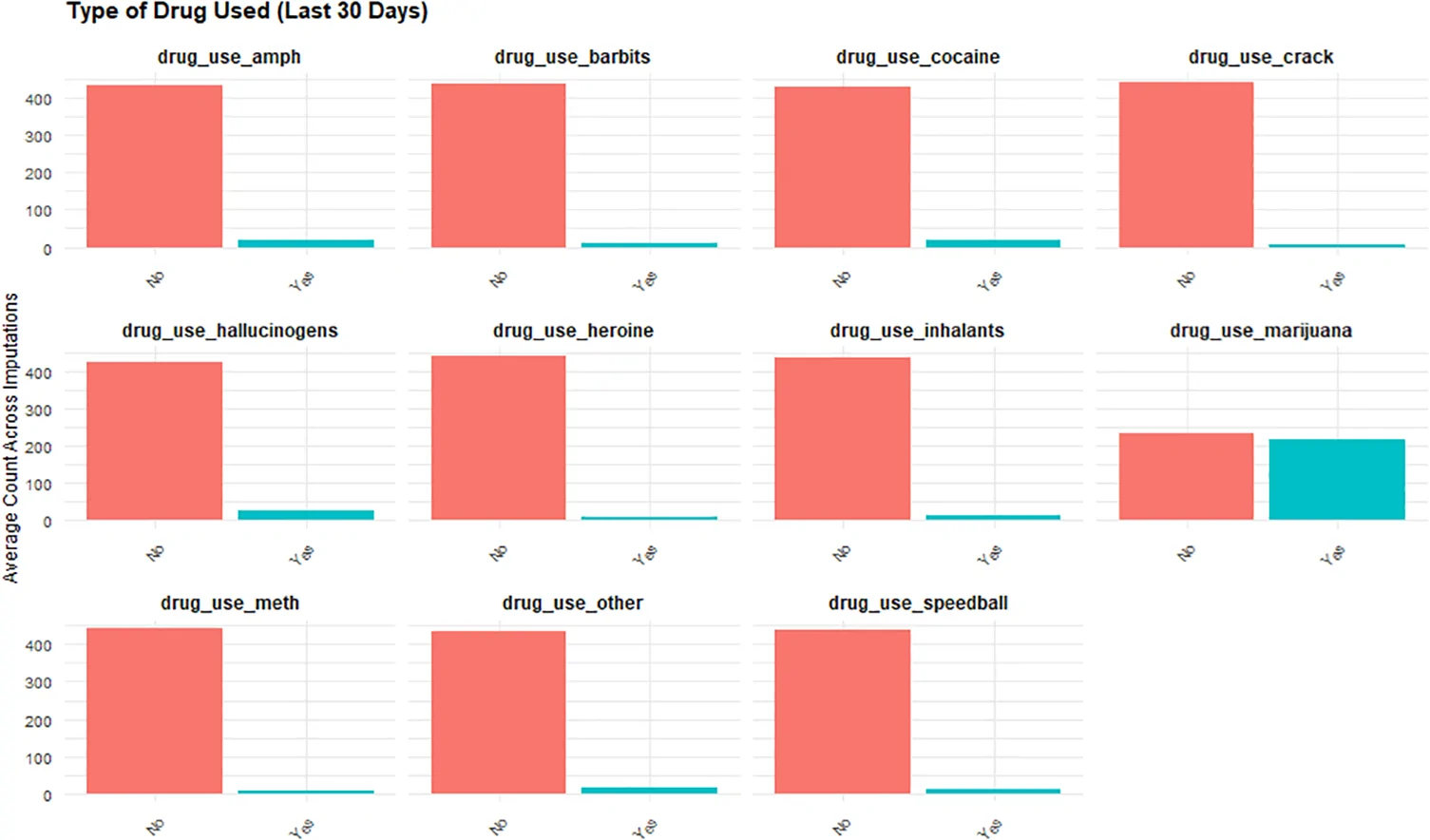
Past-30-day substance use among youth experiencing homelessness in Houston, Texas. Percentages represent the proportion of participants reporting use of each substance during the previous 30 days.

Based on the responses, it was also found that around 27.4% of the participants reported experiencing sexual assault, while 23.6% reported as having traded for sex in their lifetime. Types of drug use is presented in Fig 1. Nearly one half (48.2%) of participants reported using Marijuana in the past 30 days. Use of hallucinogens, inhalants, crack, speedball, heroin, cocaine, methamphetamine, amphetamine, and barbiturates was low, ranging from 1.6% to 5.5%.

### Bivariate analysis

Results from the univariate Modified Poisson regression analysis are presented in Table 2. Among respondents who self-identified as having experienced sex trafficking, 63.5% identified as female, 59.6% reported some form of drug use in the previous 30 days, and 60.6% identified as Black. Gender was found to be a statistically significant predictor, with females (PR = 2.53, 95% CI 1.682–3.812) and other gender (PR = 2.12, 95% CI 1.050–4.293) having higher prevalence of experiencing sex trafficking (PR = 1.69, 95% CI 1.171–2.426). Participants with a history of foster care had increased prevalence (PR = 1.53, 95% CI 1.067–2.189) of experiencing sex trafficking compared to those with no foster care experience. Lastly, having a last permanent residence within Houston had lower prevalence of experiencing sex trafficking compared to someone who did not have their residence within Houston; however, this association was not found to be statistically significant (PR = 0.74, 95% CI 0.523–1.045). Age, drug use in the last month, and race/ethnicity were found not to have significant bivariate associations with sex trafficking.

**Table 2:** Bivariate associations with sex trafficking.

Variable	Experienced Sex Trafficking		Unadjusted PR (95% CI)	p-values
	No (n = 346)	Yes (n = 104)		
**Age**	21 (4)	21 (4)	0.99 (0.911, 1.076)	0.819
				
**Gender** **Male** **Female** **Others**	196 (86.7%) 129 (66.2%) 21 (72.4%)	30 (13.3%) 66 (33.8%) 8 (27.6%)	* 2.53 (1.682, 3.812) 2.12 (1.050, 4.293)	< 0.001 0.036
**Identifies as LGBTQ+** **No** **Yes** **Not Sure**	224 (81.8%) 98 (68.5%) 24 (72.7%)	50 (18.2%) 45 (31.5%) 9 (27.3%)	* 1.69 (1.171, 2.426) 1.44 (0.075, 2.780)	0.005 0.274
**History of Foster Care** **No** **Yes**	241 (80.3%) 105 (70.0%)	59 (19.7%) 45 (30.0%)	* 1.53 (1.066, 2.189)	0.021
**Drug Use (in past 30 days)** **No** **Yes**	164 (79.6%) 182 (74.6%)	42 (20.4%) 62 (25.4%)	* 1.22 (0.822, 1.823)	0.318
**Houston Residency** **No** **Yes**	117 (72.2%) 229 (79.5%)	45 (27.8%) 59 (20.5%)	* 0.74 (0.523, 1.045)	0.087

***Note. Percentages represent the row percentages within each level of predictors***

* *Reference categories*

### Adjusted Analysis

Results from the multivariable modified Poisson regression analysis are presented in Table 3. After adjusting for other predictors, female study participants had higher prevalence than male participants of experiencing sex trafficking (aPR = 2.341, 95% CI 1.530–3.582). Age, ethnicity, sexual orientation, drug use, Houston residency, and history of foster care were not statistically significant predictors, although foster care approached statistical significance (*aPR = 1.416, 95% CI 0.990, 2.025)*.

**Table 3:** Associations with sex trafficking in adjusted analysis.

Variable	Adjusted PR (95% CI)	P-value
**Age**	0.999 (0.924, 1.079)	0.976
**Ethnicity/Race (Ref: White)** *Black* *Hispanic or Latino* *Others (incl. Mixed)*	1.372 (0.708, 2.662) 1.732 (0.784, 3.826) 1.492 (0.720, 3.095)	0.349 0.174 0.282
**Gender (Ref: Male)** *Female* *Other*	2.341 (1.530, 3.582) 1.817 (0.850, 3.882)	< 0.001 0.123
**Identifies as LGBTQ+ (Ref: No)** *Yes* *Not Sure*	1.339 (0.904, 1.985) 1.383 (0.731, 2.617)	0.145 0.319
**History of Foster Care**	1.416 (0.990, 2.025)	0.057
**Drug Use (in the last month)**	1.361 (0.836, 1.845)	0.282
**Houston Residency**	0.747 (0.535, 1.043)	0.087

## Discussion

Nearly one in four YEH in this sample reported experiences consistent with sex trafficking, highlighting the substantial burden of exploitation among youth accessing homelessness services in Houston. Female-identifying youth were disproportionately affected, with significantly higher prevalence of reporting experiences consistent with sex trafficking than male-identifying youth. Our findings are consistent with prior research, that cisgender women are disproportionately represented in detected cases of sex trafficking among YEH in the U.S. [15,24,25]. While the current findings generally align with global patterns [26,27], reliance on current methodologies likely underestimates trafficking by gender across YEH sub-groups [24,26–28].

In our study, having a history of foster care involvement was not statistically significant in the adjusted analysis; however, evidence from prior studies indicate youth with prior foster care involvement are at an increased risk of sex trafficking [14,29]. Homelessness, along with other factors such as abuse history and juvenile justice involvement, have been shown to contribute to this vulnerability in other samples [24]. This study may not have found a statistically significant association because we only measured the history of foster care, while other studies documented that the length of time or number of placements in foster care were associated with increased vulnerability to experiencing sex trafficking [18,29]. Race was not a significant predictor of sex trafficking, which is inconsistent with recent studies that have reported that a substantial proportion of people experiencing homelessness have been from the Black and Hispanic communities [15,30].

Youth who identified as LGBTQ+ in our sample showed a higher prevalence in bivariate analysis, although this association was not statistically significant in the adjusted model. This pattern may reflect overlapping vulnerabilities well-documented in the literature, including stigma, family rejection, discrimination, homelessness, and survival sex. LGBTQ+ youth experience disproportionate rates of homelessness and are significantly more likely to engage in sex exchange to meet basic needs such as food, shelter, or safety [31,32]. Among YEH, LGBTQ+ individuals have been found to have more than twice the prevalence of being sex trafficked compared to heterosexual peers and to report higher rates of prior sexual assault, substance use, and suicide attempts [31]. Similarly, research among LGBTQ+ youth in out-of-home care highlights survival sex as a response to intersecting social exclusion, economic precarity, and systemic transphobia [32]. Research also emphasizes that LGBTQ+ individuals, especially those facing homelessness or migration, remain among the most hidden victims of sex trafficking globally due to stigma and underreporting [33]. The decrease in the association in the adjusted model likely reflects confounding effects due to other social determinants, such as gender and foster care involvement, both of which disproportionately affect LGBTQ+ youth. These findings reinforce the need for inclusive, trauma-informed, and affirming services that address the compounded risks of sexual, and gender minoritized youth within homelessness and child welfare systems.

Being a Houston resident (last permanent housing was in Houston) was not associated with experiencing sex trafficking. It is possible that the last permanent housing did not capture the migratory nature of the YEH in this sample. Substance use was also not associated with being sex trafficked, even though the relationship between substance uses and sex trafficking has been documented in various ways, including playing a role in survival sex, coercive sex, and sexual trauma [25,34]. Additional research is needed in this area to examine the potential for increased vulnerability of YEH for risky behaviors, including substance use, substance use during sex, and need-based sex [34]. Migratory YEH may be more disconnected from social relationships and resources, leaving them more vulnerable to being sex trafficked.

Our findings suggest that nearly one in four YEH have experienced sex trafficking. This underscores the urgent need for systems of care, including homeless shelters and service providers, to implement standardized intake protocols that screen for human trafficking. Evidence from a Houston shelter shows that using a trafficking-specific screener identifies substantially more sexual and labor exploitation, and key risk factors, than routine psychosocial face-to-face assessments [13]. The Urban Institute’s Human Trafficking Screening Tool (HTST), designed for youth involved in child welfare and homeless youth services, is one of several tools recommended for these settings [13]. Accordingly, we encourage adoption of the HTST as part of service intake workflows. Service providers across shelters, drop-in centers, and health care settings should also be trained to recognize indicators of exploitation and to respond with trauma-informed care. Trauma-Focused Cognitive Behavioral Therapy (TF-CBT) has demonstrated feasibility and clinically meaningful reductions in PTSD symptoms when embedded within shelter services for YEH [35].

Beyond service-level changes, prevention outreach should extend to transportation hubs, transitional housing, and migration corridors where youth may be disconnected from protective networks, and into digital spaces. Young people frequently encounter and seek health information on social platforms (e.g., Facebook, YouTube, Instagram), suggesting these channels can be leveraged for outreach and education [36]. At the same time, social media is documented as a venue for trafficking recruitment and communication; hotline data show recruitment across mainstream platforms (e.g., Facebook, Instagram, Snapchat), while survivors report using social media both during exploitation and to reconnect with support [37]. Prevention and treatment outreach strategies should therefore pair digital safety messaging with credible, youth-centered content in the spaces YEH already uses [38].

Policy efforts should prioritize prevention interventions that address transitions out of foster care, histories of sexual violence, and gaps in housing and social services, especially for youth that are new to a community. Research has also highlighted provider bias in their responses to trafficking manifests at multiple levels, through stereotyping and structural barriers, and underscores the urgent need for holistic interventions in prevention, treatment, research, and policy [39]. Future research should employ longitudinal and advanced modeling approaches, such as structural equation modeling, to better understand causal pathways and mediating factors in sex trafficking. Importantly, researchers should disaggregate data to reveal patterns of marginalization and ensure that prevention and treatment interventions are tailored to the specific realities of diverse youth.

### Strengths and Limitations

By recruiting a large and diverse sample of YEH from multiple service sites in Houston, TX, this study captured a wide range of lived experiences, including marginalized groups who are often underrepresented in research yet overrepresented among YEH. The use of validated measures and rigorous analytic approaches further strengthens the reliability of the findings and provides valuable insights to inform practice, policy, and future research on trafficking among YEH. At the same time, certain limitations should be considered when interpreting the results. First, the cross-sectional design precludes any inference of causality between sex trafficking and the associated demographic or psychosocial factors identified. Second, although the survey was conducted during the COVID-19 pandemic, this analysis did not examine the pandemic’s potential influence on financial instability, housing precarity, or exploitation, all of which may have shaped youth experiences. Finally, although this study enrolled street-dwelling youth who were disconnected from services, the use of convenience and snowball sampling from shelters and drop-in centers may limit generalizability to youth outside formal service networks, including those who remain unsheltered or hidden from care systems. This may have also affected the statistical power of the analysis to detect modest associations. Additionally, there is a possibility of selection bias, which could not be addressed in the analysis. Eligibility was limited to English-speaking participants, potentially excluding experiences of youth with limited English proficiency who may face distinct trafficking vulnerabilities. Furthermore, the study relied on a specific homelessness definition derived from McKinney-Vento criteria and did not include all youth who might qualify under alternative definitions of homelessness or imminent housing instability. As a result, some vulnerable youth populations may not be represented in the findings.

## Conclusion

In this cross-sectional study of YEH in Houston, Texas, nearly one in four participants reported experiences consistent with sex trafficking, with significantly higher prevalence among female-identifying youth. These findings highlight the profound influence of structural inequities, including unstable housing, gender-based vulnerability, and limited access to supportive services, on risk for exploitation. Efforts to address trafficking among YEH should operate across structural, organizational, and interpersonal levels. Practice and policy should prioritize routine, trauma-informed screening using validated tools in shelters, drop-in centers, and healthcare and social service settings. Furthermore, service providers should be equipped to identify, engage, and support youth at risk for or with histories of exploitation through coordinated, survivor-centered approaches that strengthen pathways to safety, stability, and care.
